# Bronchial Dieulafoy’s disease: a rare but fatal condition

**DOI:** 10.36416/1806-3756/e20250457

**Published:** 2026-05-22

**Authors:** Xianping Huang, Bin Zeng, Binglin Lai

**Affiliations:** 1. Department of Medical Imaging, Ganzhou Hospital of Traditional Chinese Medicine, Ganzhou, People’s Republic of China.; 2. Department of Medical Imaging, The Fifth People’s Hospital of Ganzhou, Ganzhou, People’s Republic of China.; 3. Ganzhou Institute of Medical Imaging, Ganzhou Key Laboratory of Medical Imaging and Artificial Intelligence, Medical Imaging Center, Ganzhou People’s Hospital, Ganzhou Hospital-Nanfang Hospital, Southern Medical University, Ganzhou, People’s Republic of China.

**Figure 1:**
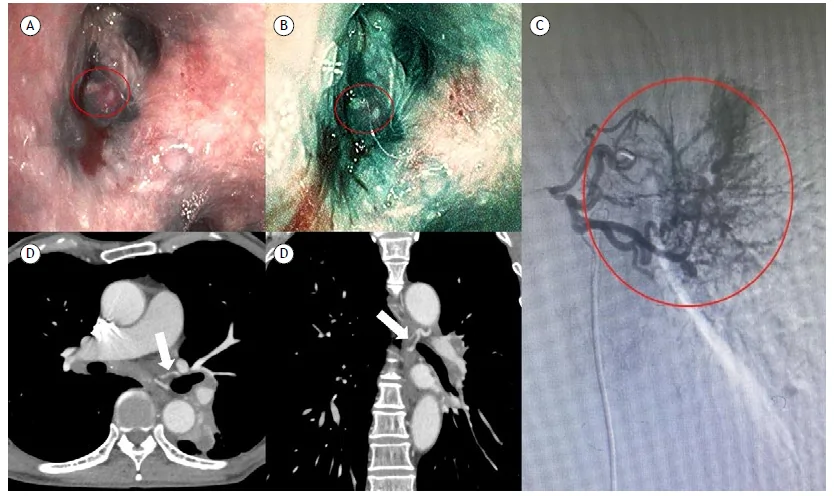
In A, white-light bronchoscopy revealing a congested, tumor-like protrusion (red circle) in the proper segment of the upper lobe. In B, narrow-band imaging revealing dilated submucosal vessels appearing in dark bluish green (red circle) and corresponding to the smooth protrusion seen under white light; by enhancing mucosal-vascular contrast, narrow-band imaging supports the diagnosis of submucosal arterial malformation. In C and D, axial and coronal contrast-enhanced CT scans showing the dilated bronchial artery (arrows) protruding into the bronchial lumen. In E, digital subtraction angiography showing the catheter tip just entering the main trunk of the bronchial artery, with the filled vascular malformation cluster (red circle) visible at the instant before embolization.

A 72-year-old male presented with 3-day symptoms of chest tightness and cough, plus a history of hemoptysis. His risk factors included a 30-year smoking history and 8 years of mine dust exposure. Chest CT revealed diffuse pulmonary silicosis and left lower lobe bronchial stenosis. Bronchoscopy identified a congested, tumor-like protrusion in the upper lobe (Figure 1A). Under narrow-band imaging, the lesion displayed thick, tortuous vessels appearing in dark bluish green (Figure 1B) and indicating that a submucosal artery curved directly to the surface-a characteristic finding of bronchial Dieulafoy’s disease (BDD). To avoid life-threatening hemorrhage, no biopsy was performed. Contrast-enhanced CT scans (Figures 1C and 1D) further confirmed BDD. The patient was successfully treated with bronchial arterial embolization via digital subtraction angiography (Figure 1E) and had no hemoptysis recurrence after two years. 

BDD is a rare, potentially fatal bronchial submucosal vascular malformation. It is characterized by an abnormally large submucosal artery that lacks a normal capillary network; its rupture can cause recurrent or massive hemoptysis.^1^ ^,^ ^2^ Bronchoscopy is the definitive diagnostic procedure. The endoscopic finding of smooth, nodular protrusions should raise immediate suspicion of BDD. Crucially, blind biopsy must be avoided to prevent the risk of fatal hemorrhage. 
